# Proapoptotic genes BAX and CD40L are predictors of survival in transitional cell carcinoma of the bladder

**DOI:** 10.1038/sj.bjc.6600765

**Published:** 2003-02-18

**Authors:** S A Hussain, R Ganesan, L Hiller, P G Murray, M M El-Magraby, L Young, N D James

**Affiliations:** 1 Cancer Research UK Institute For Cancer Studies, University of Birmingham, Vincent Drive, Edgbaston, Birmingham B15 2TT, UK; 2 Department of Pathology, Birmingham Woman's Hospital, Birmingham, UK; 3 Cancer Research UK Trials Unit, Institute For Cancer Studies, Birmingham, UK

**Keywords:** bladder cancer, apoptosis, prognostic markers, BAX, CD40L

## Abstract

The purpose of the study was to investigate the effects of expression of a range of genes involved in apoptosis on outcome in bladder cancer. Immunohistochemistry was used to examine expression of BCL2, BAX, P53, CD40 and CD40L in archival tissues of patients included in various treatment trials for transitional cell carcinoma (TCC) of the bladder. Data were collected on 94 patients who first presented with either invasive or superficial bladder cancer. Median follow-up for alive patients was 83 months (m) (range 12–195 m). Median survival was 80 m (95% CI=56–128 m). Median survivals for the various markers were as follows: BAX-positive patients 110 m *vs* BAX-negative patients18 m (*P*=0.0002); CD40L-positive patients 95 m *vs* CD40L-negative patients 45 m (*P*=0.04); BCL2-positive patients 44 m and BCL2-negative patients 74 m, (*P*=0.64); CD40-positive patients 110 m and CD40 negative patients 45 m (*P*=0.12); and P53 positive patients 80 m and P53 negative patients 45 m (*P*=0.58). In conclusion, it was seen that overexpressions of BAX and CD40L are prognostic of better survival in TCC of the bladder. Our results also raise the possibility of the future development of CD40- and CD40 ligand-based immunotherapy for bladder cancer. This study links proapoptotic and antiapoptotic markers to overall survival.

Bladder cancer will be diagnosed in more than 54 000 people this year in USA, and over 12 000 will die of the disease (ASCO online). Bladder cancer, with 12 730 new cases in 1997, is the fifth commonest cause of cancer in the UK (Cancer Research UK Fact Sheet). Approximately 20% of newly diagnosed cases are invasive and, in this group, prognosis is poor with only 60% of T2 and 40% of T3 patients surviving 5 years. Patients with more advanced or nodal disease do significantly worse; median survival with metastatic disease for those patients fit enough to enter trials is approximately 13 months (m). Management of locally advanced disease is by surgery or radiotherapy, both of which have significant drawbacks. Neoadjuvant and adjuvant chemotherapy have been tested extensively but to date this approach has not had a significant impact on overall survival (Frieha *et al*, 1996; Malmstrom *et al*, 1996; Stockle *et al*, 1996; International Collaboration of Trialists, 1999). Cisplatinum-based combination chemotherapy forms the mainstay of therapy for relapsed or metastatic disease with response rates of up to 60–70% reported, but few long-term survivors. Intravesical and systemic chemotherapy are limited in their efficacy in the treatment of bladder cancer patients as they are unable to induce apoptosis in bladder tumour cells. Understanding the apoptotic signals and the cascade of reactions that give prosurvival signals will go a long way in refining the treatments and will help in future to individualise cancer therapies.

We studied a range of molecular markers to define their roles and their association with other markers to define targets against which we can target appropriate therapies.

CD40 is a 48 kDa membrane glycoprotein of the tumour necrosis factor receptor (TNFR)/nerve growth factor superfamily, and is expressed on the surface of B lymphocytes, dendritic cells, epithelial and haemopoietic stem cells (Lee *et al*, 1997), and various tumours. It was first described in 1985 as a bladder tumour antigen (Banchereau *et al*, 1994) but this was largely overlooked when subsequent investigation revealed its pivotal role in B-cell function, where ligation with CD40 ligand (CD40L or CD154) expressed on activated T-helper cells stimulates cell maturation, proliferation and immunoglobulin class switching. Despite its role as a promoter of B-cell growth, it has been previously shown that, in common with other members of the TNFR family, CD40 stimulation sensitises carcinoma cells *in vitro* to apoptosis induced by various stimuli including Fas, ceramide and cytotoxic chemotherapy (Eliopoulos *et al*, 1996).

CD40 ligand (CD40L), also known as CD154, functions as the natural ligand for CD40 (Spriggs *et al*, 1992; Armitage *et al*, 1992). It is expressed primarily by on the surface-activated T lymphocytes (Armitage *et al*, 1992) but has also been found on activated T platelets (Henn *et al*, 1998). Interactions between CD40 and CD40L provide critical costimulatory signals that trigger T-lymphocyte expansion (Grewal *et al*, 1998).

We have previously demonstrated that quantification of the antiapoptotic protein BCL2 in patients undergoing neoadjuvant chemotherapy plus radiotherapy for advanced bladder cancer may identify patients who might benefit from neoadjuvant chemotherapy (Cooke *et al*, 2000). The BCL2 oncoprotein inhibits apoptosis and is overexpressed by many tumours including breast (Hellemans *et al*, 1995), colon (Bronner *et al*, 1995), prostate (Krajewska *et al*, 1996) and tumours of the head and neck (Gallo *et al*, 1999). By virtue of its biological activity, it may be associated with a poor prognosis, with resistance to current treatment modalities including radiotherapy, for example, in cervical (Harima *et al*, 1998) and prostate cancer (Apakama *et al*, 1996). The data available to date, describing the role played by BCL2 in transitional cell carcinoma (TCC) of the bladder, have been both limited and conflicting, with some studies showing an association with a lower tumour grade (King *et al*, 1996) and less aggressive phenotype (Shiina *et al*, 1996). Others have shown the reverse, with expression being greater in higher-stage and higher-grade tumours, resulting in increased frequency of disease recurrence and higher disease progression rates, leading to shortened survival (Ye *et al*, 1998; Pollack *et al*, 1997; Kong *et al*, 1998). It is likely that these conflicting results arise because of different interactions between the various treatment modalities and apoptotic pathways. Many of the effects of BCL2 depend on its ratio to BAX as well as on its absolute level. The proapoptotic BH-123 proteins share three of the BCL2 homology (BH) domains with the antiapoptotic proteins and include BAX, BAK, and BOK. BAX plays a central role in regulation and commitment to apoptosis. In order to further examine these phenomena, we therefore set out to investigate the linked genes BAX and BCL2 expression in the same group of patients, and to examine whether these discrepant results are explained by variations in other genes involved in the same pathways.

We therefore proceeded with immunohistochemical analysis of these anti-apoptotic and pro-apoptotic genes to look for correlation between themselves and the effects of their expression on clinical outcome and survival. We report the expression of antiapoptotic BCL2, proapoptotic BAX, CD40, CD40L and accumulation of P53 in a series of patients with transitional cell carcinoma of the bladder.

## MATERIALS AND METHODS

Expression of genes was carried by immunohistochemical analysis of the paraffin tissue archive. The tissue collection and the study had full local research ethics approval. Ninety-four patients with TCC of the bladder were identified from hospital records (74 males, 20 females, median age 67 years, range 32–89 years). Of these, 45 had noninvasive tumours and were included in two clinical trials for primary and recurrent Ta and T1 tumours, and 49 had invasive tumours, of which 26 patients were treated in a pilot study of chemoradiation in muscle-invasive bladder cancer (Hussain *et al*, 2001) and 23 patients were treated in a radiotherapy study examining the role of neoadjuvant cisplatin for muscle-invasive tumours (Wallace *et al*, 1991). Histological classification of the patients' tumours was obtained from the original hospital/trial pathology records and was according to the Union Internationale Contre le Cancer (UICC) TNM system, 1997. The archived tissue specimens were retrieved, all of which had been routinely fixed in 10% saline-buffered formalin and embedded in paraffin wax. One representative tissue block was chosen for each patient on the basis of gross specimen morphology, and serial 5 *μ*m sections were cut from it and mounted onto microscope slides. One section from each specimen was then immunostained for BCL2, BAX, CD40, CD40L and P53, plus a specimen for routine haematoxylin and eosin staining.

### Staining procedure

Tissue sections were deparaffinised in xylene, washed in alcohol and rehydrated in 0.05 MpH 7.6 phosphate-buffered saline (PBS). Antigen retrieval was performed in a 700 W microwave oven in 0.01 M pH 5.8 citrate buffer solution for 30 min as optimised in preliminary studies. The sections were cooled to room temperature in PBS, the primary antibody applied and incubated in a moist chamber at room temperature for 1 h. The primary antibodies and concentrations used were as shown in Table 1

**Table 1:** Antibodies used

**Antibody**	**Code**	**Source**	**Dilution**	**Supplier**
BCL2	(C-2):Sc-7382	Mouse monoclonal antibody	1 : 10	Santa Cruz, CA, USA
BAX	P-19:Sc-526	Rabbit purified polyclonal antibody	1 : 20	Santa Cruz, CA, USA
CD40	(N-16):Sc-974	Rabbit polyclonal antibody	1 : 100	Santa Cruz, CA, USA
CD40L	(C-20):Sc-978	Rabbit polyclonal antibody	1 : 100	Santa Cruz, CA, USA
P53	(Bp 53-12):Sc-263	Mouse monoclonal antibody	1 : 20	Santa Cruz, CA, USA

. The secondary staining and tertiary staining was performed using DAKO strept ABC complex (DAKO Ltd, Cambridgeshire, UK). Secondary and tertiary antibodies were applied and incubated in a moist chamber at room temperature for 30 min. Sections were counterstained in Mayer's haematoxylin and mounted in aqueous mountant (Shandon, UK). Sections of tonsil were used as positive control for each staining run. Substituting primary antibody with PBS created negative controls. Tissue lymphocytes acted as an additional internal control for the CD40- and BCL2-stained sections. Evaluation of immunostaining (Table 2

**Table 2:** Evaluation of staining

	**BCL2, BAX, CD40, CD40L**	**P53**
Diffuse	Confluent positivity	30% of nuclei
Focal	Nonconfluent islands of positivity	<30% of nuclei
Strong	Intensely brown	Intensely brown
Weak	Weakly brown	Weakly brown

) was performed on two separate occasions by one observer (SAH) and once by a pathologist (RG), both of whom were blinded to any other data. The whole of each section was subjectively assessed under light microscopy. There was one score for the strength of staining (absent, weak or strong, respectively, as compared with the positive controls) and one score for the percentage of tumour stained (absent, focal/patchy or diffuse). Staining of mitotic figures was ignored and only nuclear staining was regarded as positive for P53. Sections where intra or interobserver error occurred for either of the scores were reviewed again by a pathologist (RG or MM) and assigned a score that dictated which of the two original scores was recorded. In the event of all three pairs of scores differing, a consensus score was agreed upon after examination under a multiheaded microscope. Tumours were classified into five patterns of expression: tumour negative; weak diffuse (WD); weak focal (WF); strong diffuse (SD); or strong focal (SF). Of the 94 specimens, satisfactory staining was achieved in 86 cases (91.5%). In cases where staining was not optimal for evaluation, they were left out of the study. In some cases only normal or inflammatory tissue was identified with no evidence of transitional cell carcinoma. In the remaining 81 out of the 94 (86%) cases stained for BAX, 83 out of the 94 (88%) for BCL2, 79 out of the 94 (84%) for CD40, 79 out of the 94 (84%) for CD40L and 78 out of the 94 (83%) for P53 contained foci of malignancy.

### Statistical methods

Intercorrelations between the laboratory measures were explored by use of Fisher's exact tests and Correspondence Analysis (Greenacre, 1984), an exploratory technique used to analyse multi-way tables. Survival times were calculated as the date of primary tumour diagnosis to date of death, or date of censor if alive. Survival curves were constructed using the method of Kaplan and Meier (1958), and the log-rank test (Peto *et al*, 1977) was used to assess any differences between patient and tumour characteristics. Cox proportional hazards analysis (Cox, 1972) was also performed, using a 5% entry and exit criterion, to determine important independent prognostic factors of survival.

Statistical analysis was carried out independently using SAS statistical software (SAS Institute, Cary, NC, USA).

## RESULTS

### Intercorrelation between laboratory measures

Fisher's exact tests showed the BCL2 marker to be independent of all others (*P*>0.05). P53 was also shown to be independent of all other markers, except a borderline dependence with BAX (*P*=0.04). BAX, CD40 and CD40L were all significantly associated with each other (*P*⩽0.05). Correspondence analysis confirmed the above by showing a separate tight clustering of the expression levels of BAX, CD40 and CD40L, which were also separate from the two distinct levels of both BCL2 and P53. This indicates that BAX, CD40 and CD40L vary together and thus explain similar characteristics about patients. BCL2 and P53 are shown as individual markers dissimilar to all others thus measuring different things.

**Figure 1:**
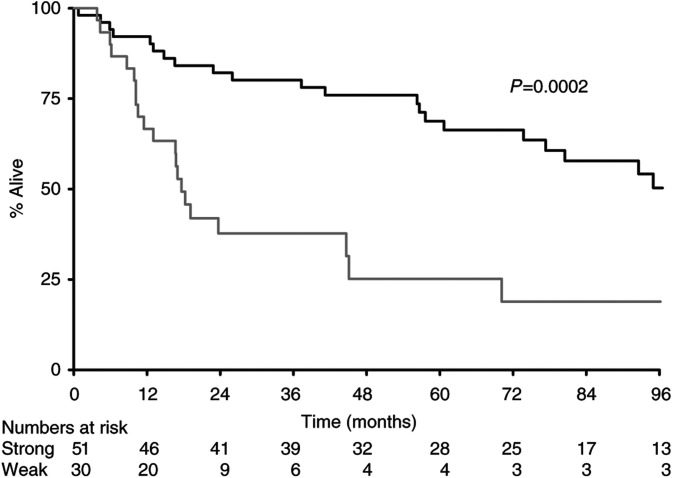
Survival by BAX (**---** strong; — weak).

Analysis of the individual subgroups (negative, WF, WD, SF, SD) demonstrated statistically significant interactions between all subgroups of expression for BAX, with strong positivity with a diffuse pattern of BAX expression as an independent prognostic marker of survival (*P*=0.0001). However, since focality of expression was a relatively subjective observation dependent on variable factors such as the even distribution of antigen at the time of staining, we grouped the results into better definable groups based only on the strength of staining. Despite ignoring the pattern of staining, BAX remained an independent marker of prognostic significance of survival (*P*=0.0002). Patients with tumours overexpressing BAX had a median survival of 110 m as against a group of patients with weak expression or no expression of BAX who had a median survival of 18 m (Figure 1

). Similar considerations applied to CD40L and so, for practical purposes, cases were then grouped as either positive (strong) for (SF or SD) expression or negative (weak) for (negative, WF or WD) expression, as this gave the most easily clinically applicable scoring system. There were no significant differences seen for expression of CD40, P53 or BCL2, but the same grouping system was applied to these groups in the interests of consistency of analysis.

**Figure 2:**
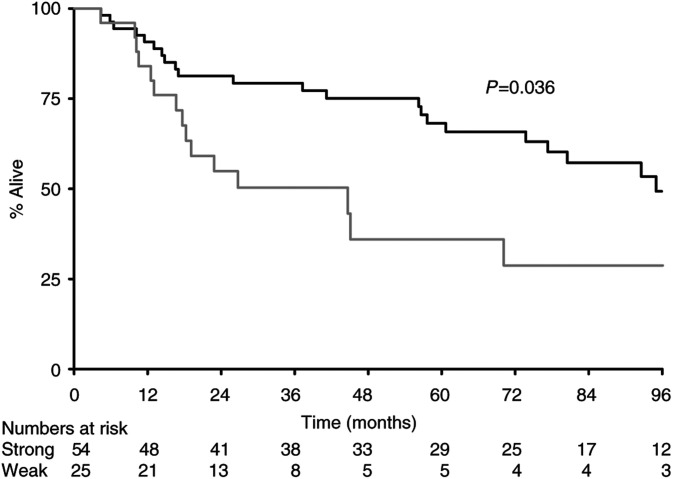
Survival by CD40L (**---** strong; — weak).

**Figure 3:**
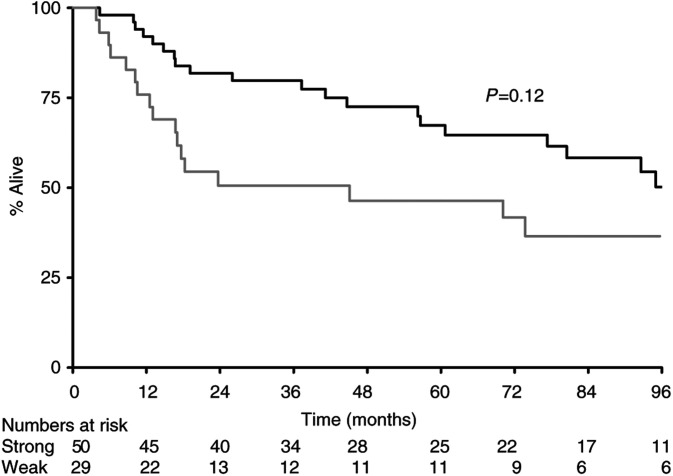
Survival by CD40 (**---** strong; — weak).

**Figure 4:**
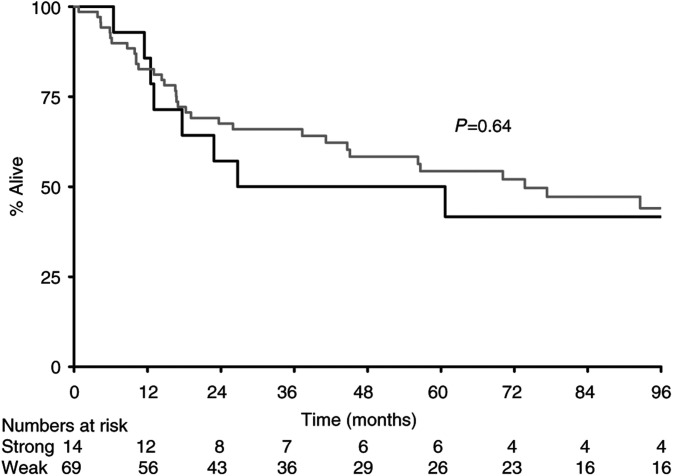
Survival by BCL2 (**---** strong; — weak).

**Figure 5:**
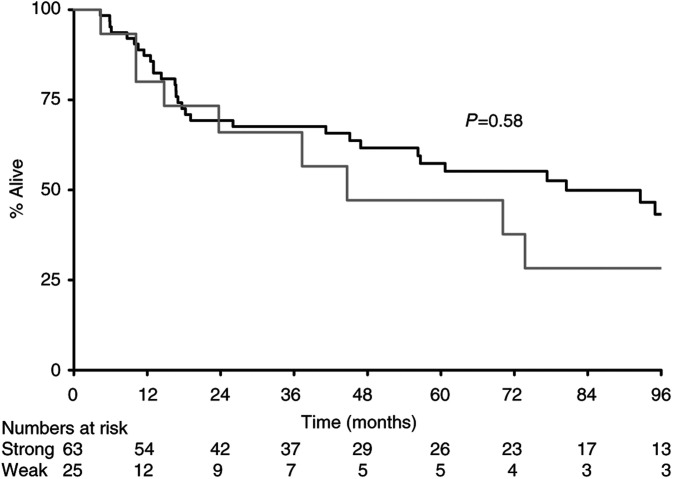
Survival by P53 (**---** strong; — weak).

**Figure 6:**
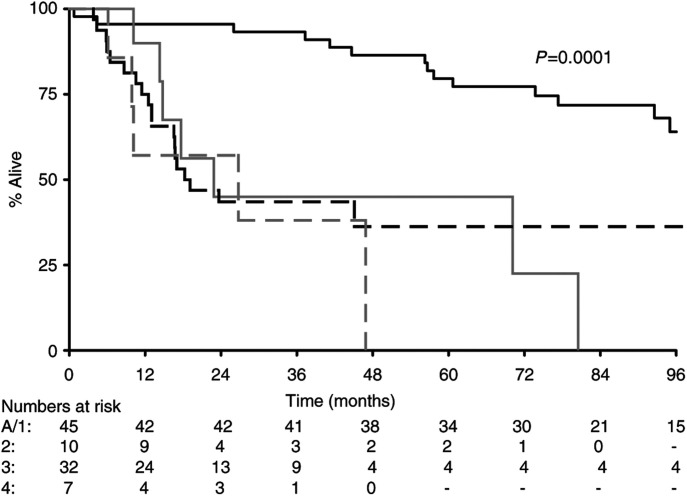
Survival by stage (**---** stage A/1; — stage 2; **--** **--** stage 3; – – stage 4).

**Figure 7:**
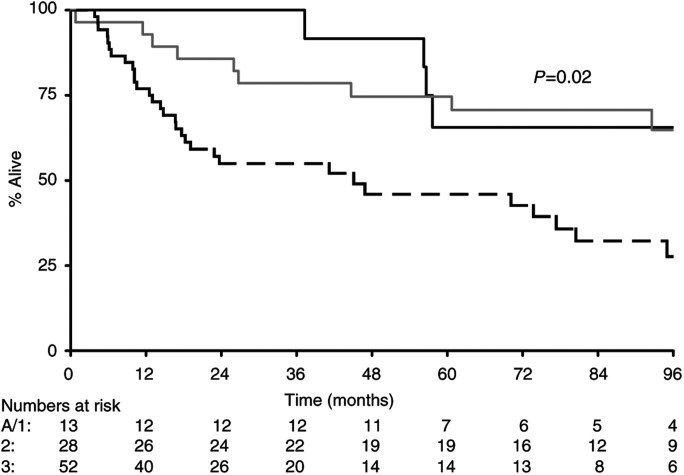
Survival by grade (**---** grade 1; — grade 2; – – grade 3).

The median survival for all patients from the day of diagnosis of primary tumour was 80 m. There were statistically significant effects according to expression levels of BAX (110 m *vs* 18 m, *P*=0.0002) (Figure 1) and CD40L (95 m *vs* 45 m, *P*=0.04), with overexpression of CD40L leading to better survival (Figure 2

). For CD40, median survival by subgroup was positive 110 m and negative 45 m, *P*=0.12 (Figure 3

); for BCL2, positive 44 m and negative 74 m, *P*=0.64 (Figure 4

). For P53, the results were again not significant with median survivals of 80 m for positive and 45 m for negative *P*=0.58 (Figure 5

). As would be expected, there were significant associations between tumour stage (Ta/1 *vs* T2 *vs* T3 *vs* T4 with median survivals of 128, 23, 19 and 27 m, respectively, *P*=0.0001) (Figure 6

) and grade (grade 1 *vs* grade 2 *vs* grade 3: 111, 128 and 45 m, respectively, *P*=0.02) (Figure 7

).

### Cox regression on survival

Cox regression showed stage (Ta or T1 *vs* T2–4) to be the most important independent prognostic factor (*P*<0.0001, HR=1.77, 95% CI 1.34–2.33) and, if it is known, no other variable adds to the accuracy of prognosis. However, if stage is unknown, BAX (*P*=0.0003, HR=3.00, 95% CI=1.64–5.48) and CD40L (*P*=0.04, HR=1.99, 95% CI=1.03–3.83) are shown to be important prognostic markers although either of the one rules out the need for the other. No further variables were deemed important in survival prognosis.

## DISCUSSION

A fundamental difficulty in the understanding, prevention and treatment of cancer is that the currently recognised disease classes are each really a collection of diseases having significant features in common (e. g. the organ where the tumour arose), but also many features that distinguish them. The diversity within most disease categories is reflected in a diversity of responses to specific therapeutic regimens. Numerous studies indicate that this clinical heterogeneity reflects underlying molecular heterogeneity, and therefore we have begun to use gene expression measurements to build a higher resolution and more clinically relevant taxonomy of human tumours. Our study analysed a large collection of archived tumour samples, where the treatment history and outcomes of the patients were well documented, and reveals interesting associations with a group of genes involved in regulation of apoptosis.

The CD40 receptor is expressed in many immune cell types and is known to play a central role in both humoral and T-cell-mediated immunity, a subject of intense research interest in recent years. It is also expressed on a variety of carcinomas and may therefore be of biological significance in the development and treatment of cancer (Young *et al*, 1998; Gallagher *et al*, 2002). Our group has previously examined expression of CD40 by immunohistochemistry in TCC of the bladder and the correlation with known prognostic markers and clinical outcome (Cooke *et al*, 1999). Seventy-eight percent of the tumours were CD40 positive, with a highly significant association with both lower stage and lower grade (*P*<0.001). CD40 expression was not found to be an independent prognostic marker of survival in our previous study. We extended these previous observations by additionally assessing the expression of CD40L and P53 in this group of patients to further investigate their role as prognostic markers.

**Figure 8:**
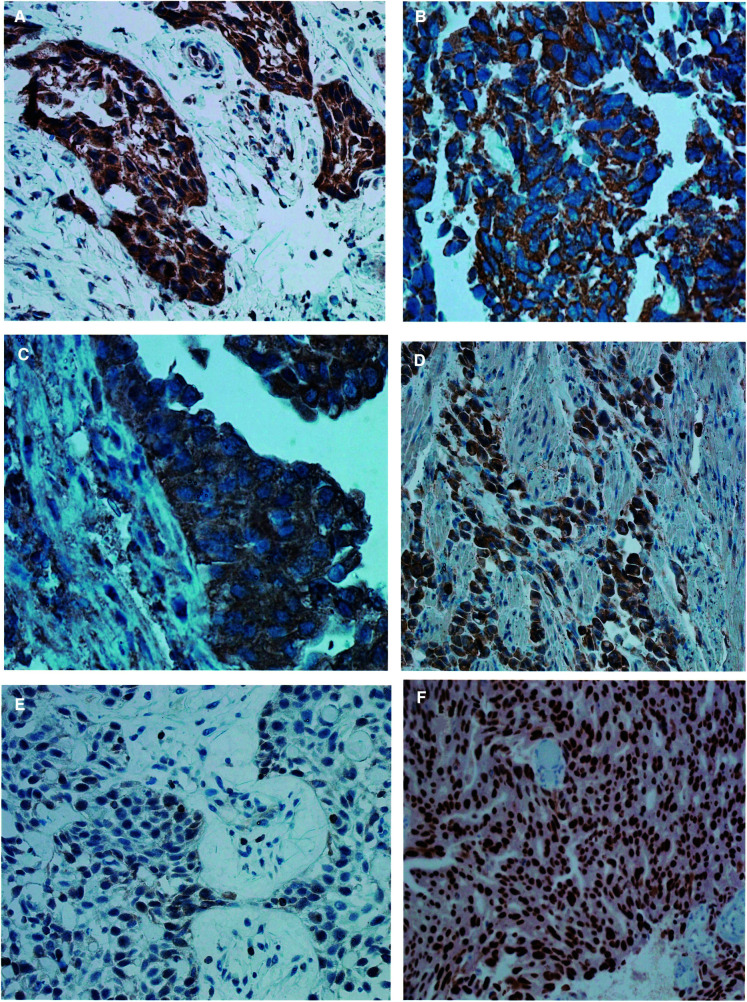
Pathology image. (**A**) Magnification×40 objective, CD40 shows diffuse strong cytoplasmic staining in myo invasive transitional cell carcinoma. (**B**)×40 objective, CD40L antibodies stains neoplastic cells strongly positive. (**C**)×40 objective, BAX shows strong positive staining of the surface in this transitional cell carcinoma. (**D**)×20 objective, shows myo invasive islands stain strongly positive for BAX. (**E**)×20 objective, weak and focal positivity of BCL2, few tissue lymphocytes serve as positive internal controls. (**F**)×20 objective, strong diffuse nuclear positivity is seen on p53 staining.

In this study, we were able to demonstrate expression of CD40L in bladder tumour tissue (Figure 8

), the first such demonstration in nonlymphoid tissue. Furthermore, overexpression of CD40L was significantly associated with improved outcome (Figure 2). Although overexpression of CD40 was not prognostically significant at the 5% level (*P*=0.12, Figure 3), it was significantly associated with expression of CD40L by Fisher's exact test, suggesting that both ligand and receptor are likely to be necessary for biological effect.

The BCL2 oncoprotein inhibits apoptosis. As a complex network of regulatory pathways control apoptosis and the sensitivity of apoptosis is the result of the balance between pro- and antiapoptotic proteins, we set out to examine other apoptosis-regulating proteins. There is increasing evidence from other tumour sites showing the role of BAX as a prognostic marker. In ovarian cancer, it was shown that high BAX expression was associated with significant improvement of the percentage of complete remissions after first-line chemotherapy with Paclitaxel and a platinum analogue. Survival was also high in groups with high BAX expression (Tai *et al*, 1998). In patients with breast cancer, loss of BAX immunostaining was associated with a decreased response to chemotherapy and shorter survival (Krajewski *et al*, 1995). In diffuse aggressive non-Hodgkin's lymphoma, reduced BAX expression was associated with a lower 8-year survival (Gascoyne *et al*, 1997). BAX plays a central role in regulation and commitment to programmed cell death. BAX counteracts the apoptosis-preventing effect of BCL2 and may directly induce apoptosis (Xiang *et al*, 1996; Zha and Reed, 1997). The proapoptotic BAX is located in the outer mitochondrial membrane (Schlesinger *et al*, 1997). BAX overexpression induces mitochondrial permeability transition, which leads to the release of cytochrome *c* (Pastorino *et al*, 1998). BAX-negative tumours, therefore, may be less susceptible to apoptosis. BAX/P53 pathway analysis in colorectal cancer with hepatic metastases showed that patients with high BAX protein expression in resected liver lesions had a median survival of 53.6 m compared with 35.4 m for patients with low BAX expression (*P*<0.05). Low BAX expression was an independent negative prognostic marker in multivariate regression analysis for all patients independent of the P53 status (Sturm *et al*, 1999). In our study, overexpression of BAX was associated with a favourable outcome (Figure 1). Expression of BAX was shown to be independent of both BCL2 and P53 both by Fisher's exact test and by Correspondence Analysis, suggesting that it relates to different aspects of the biology of the tumour. On the other hand, BAX expression was associated with expression of CD40 and CD40L, suggesting that all three are linked in their behaviours in this tumour type.

Our results also reveal a significant association between overexpression of CD40 ligand and survival. CD40 ligation thus could be used to sensitise a tumour to conventional treatment modalities such as chemo- or radiotherapy and additionally with a potential for stimulating natural immunity against solid tumours in the manner, which has already been seen in other malignancies both *in vitro* and *in vivo* (Eliopoulos *et al*, 1996; Bergamo *et al*, 1997; Dilloo *et al*, 1997; Kikuchi and Crystal, 1999). There is evidence to support the notion of CD40 ligation-induced apoptosis as a mechanism for eliminating neoplastically transformed urothelial cells (Bugajska *et al*, 2002). A phase I study of recombinant Human CD40L in patients with advanced solid tumours or intermediate- or high-grade non-Hodgkin's lymphoma demonstrated encouraging antitumour activity, including a long-term complete remission (Vonderheide *et al*, 2001). Therapeutic use of CD40L in future will be incorporated into radical treatment of bladder cancer to get a clear picture of their efficacy and toxicity. We intend to use more sophisticated techniques including microarrays in a group of patients undergoing treatment in a randomised setting, with the ultimate hope of being able to further sub-classify bladder tumours into clinically homogenous groups.

Expression of these genes was seen across the range of grades and stages of disease. Once these are stratified into various stages and grades, the number of cases decreases and the statistical power to discriminate is lost. It would be very interesting to follow patients with low-grade tumours at diagnosis in a randomised clinical trial setting with Bax, Bcl-2, CD40 and CD40L mapping and note whether overexpression or underexpression of these markers by immunohistochemistry predates the aggressive clinical behaviour and therefore indicates a point of therapeutic intervention.

In conclusion, this is the first study that has examined expression of BAX in all stages of transitional cell carcinoma of the bladder. We believe that analysis of molecular markers such as BAX, BCL2, CD40 and CD40L may prove valuable in identifying patients with poor prognosis bladder cancers who may benefit from aggressive treatment and this should be considered in future trial design. Bladder is a particularly attractive model to study, as it provides a conveniently isolated field to test new therapies, and repeated endoscopic assessment and tissue biopsies are possible during the course of treatment and post-treatment to evaluate the response to the therapy. Furthermore, the disease itself, if advanced to muscle invasion, carries a poor outlook as seen in our series, with median survival dropping from 128 m for stage Ta or T1, to 23 m for stage T2, 19 m for stage T3 and 27 m for stage T4 with a *P*-value of 0.0001 (Figure 6). This also strengthens the statement that Ta, T1 and invasive bladder cancers are two different entities with remarkably different clinical outcomes.

Endoscopic assessment of bladder tissue at various time points in organ preservation strategies will also help us to understand and answer many aspects of programmed cell death. All forms of cancer therapy risk selecting for resistance, a problem compounded by plasticity of the tumour cell genome (Green and Evan, 2002). The most effective strategy will definitely be a combination treatment attacking several targets specific to cancer. The evolution of such sophisticated forms of treatment will undoubtedly proceed by a process that employs both rational and empiric approaches. Our study generates a hypothesis linking proapoptotic and antiapoptotic markers to overall survival and needs to be tested robustly in a randomised clinical trial setting.
